# toBeeView: a program for simulating the retinal image of visual scenes on nonhuman eyes

**DOI:** 10.1002/ece3.2442

**Published:** 2016-10-11

**Authors:** Miguel A. Rodríguez‐Gironés, Alberto Ruiz

**Affiliations:** ^1^ Estación Experimental de Zonas Áridas CSIC Almería Spain

**Keywords:** acceptance angle, interommatidial angle, inter‐receptor angle, mimicry, spatial resolution, visual acuity

## Abstract

We present toBeeView, a program that produces from a digital photograph, or a set of photographs, an approximation of the image formed at the sampling station stage in the eye of an animal. toBeeView is freely available from https://github.com/EEZA-CSIC/compound-eye-simulator. toBeeView assumes that sampling stations in the retina are distributed on a hexagonal grid. Each sampling station computes the weighted average of the color of the part of the visual scene projecting on its photoreceptors, and the hexagon of the output image associated with the sampling station is filled in this average color. Users can specify the visual angle subtended by the scene and the basic parameters determining the spatial resolution of the eye: photoreceptor spatial distribution and optic quality of the eye. The photoreceptor distribution is characterized by the vertical and horizontal interommatidial angles—which can vary along the retina. The optic quality depends on the section of the visual scene projecting onto each sampling station, determined by the acceptance angle. The output of toBeeView provides a first approximation to the amount of visual information available at the retina for subsequent processing, summarizing in an intuitive way the interaction between eye optics and receptor density. This tool can be used whenever it is important to determine the visual acuity of a species and will be particularly useful to study processes where object detection and identification is important, such as visual displays, camouflage, and mimicry.

## 1 Introduction

The visual system of an animal limits the information it can extract from a visual scene and the speed and accuracy with which it can do it, shaping its behavioral responses. To understand how animals interact with the world, we must know how they perceive it.

We ignore how nonhuman animals perceive their environment, but we know a great deal about the amount and type of information to which they have access. In the first step of the visual chain, a sector of the outer world is projected onto the retina and the image formed is probed with an array of sampling stations, each estimating the flow of photons from a particular direction and in a specific range of wavelengths. The output from these stations is later processed to extract information about the shapes, colors, distances, or movement of surrounding objects.

No amount of information processing can extract information about the visual scene that the sampling stations have missed. Thus, the image projected to the sampling stations will tell us how much information the nervous system can use to see the world—regardless of how, and whether, the visual system uses the information available at this stage.

For the particular case of honeybees, *Apis mellifera*, Vorobyev, Gumbert, Kunze, Giurfa, and Menzel (1997) showed that a set of five photographs of the same image, each taken with a different band‐pass filter, could be used to simulate color perception by bees. To account for spatial acuity, they tiled the image in hexagons, each of them colored in a weighted average of the pixels projecting on the hexagon. Chiao, Wu, Chen, and Yang (2009) simulated color perception with a set of three custom‐made filters, with spectral sensitivities matching those of the bee photoreceptors, and handled spatial acuity by applying a Gaussian low‐pass filter to the image. A different approach was taken by Williams and Dyer (2007). Although their treatment of color perception was not unlike the previous ones, rather than taking high‐resolution images and processing them to simulate low acuity, they developed an optical device that directly reduced spatial resolution.

Working on these earlier developments, we have developed toBeeView: A program that extracts from a digital photograph, or a set of photographs, an approximation of the image formed at the sampling station stage in the eye of an animal observing it. toBeeView extends the tool developed by Vorobyev et al. (1997) and, most important, is easy to use and freely available to all potential users. Despite its name, its usage is not restricted to bees.

## 2 Underlying Logic

Consider first the compound eye of the honeybee *A. mellifera*. Each simple eye, or ommatidium, focuses the light impinging the eye from a region of space onto nine receptor cells (Gribakin, 1975). Bees have three photoreceptor types, maximally sensitive to light in the UV, blue, and green regions of the electromagnetic spectrum (Menzel & Backhaus, 1991). As a first approximation, we can consider that each ommatidium sends the brain information about the rate at which UV, blue, and green light is arriving from a certain direction—equating ommatidia and sampling stations. In fact, however, the bee eye is a random mosaic of three ommatidium types: Some ommatidia have UV, blue, and green photoreceptors, but other ommatidia lack either the UV or blue receptors (Wakakuwa, Kurasawa, Giurfa, & Arikawa, 2005). Because some ommatidia gather information in only two color channels, the spatial resolution of the color image at the receptor stage is lower than the one the bees would achieve if each ommatidium had UV, blue, and green receptors. We come back to this problem later.

Insects vary greatly in the number of photoreceptor types they possess and the way they are arranged in ommatidia. Many butterflies, for instance, are tetrachromatic—their eyes use four different color channels (Briscoe, 2008). *Drosophila melanogaster* has five receptor types in its retina, arranged in two types of ommatidia (Chou et al., 1999), but it has been suggested that blowflies may have a categorical color vision system, distinguishing only between four color categories (Troje, 1993) and different fly groups could have different color vision systems (Lunau, 2014). Despite this variability of detail, the main principle is retained: Eyes are composed of a random mosaic of ommatidia, each of which collects information about the amount of light arriving to the eye from a specific direction in a subset of color channels. Typically, assuming that each ommatidium collects information in all available color channels leads to overestimating the spatial resolution of the image formed at the receptor stage.

Camera eyes, characteristic of vertebrates, cephalopods, and spiders, are very different from compound eyes. Nevertheless, they also possess an array of sampling stations—photoreceptors capturing information about the flow of light in a spectral band impinging the eye from a specific direction—that provide the brain with the information from which vision emerges. An important difference between the compound eye and the camera eye is that, in compound eyes, each sampling station (ommatidium) collects information in several spectral bands. In camera eyes, however, each sampling station is a single receptor and therefore collects information in a single spectral band. Thus, while the angular spacing between ommatidia may give a reasonable indication of spatial resolution, in camera eyes, the spatial resolution of color vision is determined by the typical distance between same‐type photoreceptors. (The rods in vertebrate eyes underlie scotopic vision and play little role in color vision; we ignore them here.)

## 3 Calculations: Basic Configuration

We first consider the simplest configuration of toBeeView. We refer to this configuration as scanning mode without chromatic treatment. It is similar to the tool developed by Vorobyev et al. (1997), but it ignores the problem of color perception. In this configuration, toBeeView first superposes a hexagonal grid on the photograph, assigning one hexagon to each sampling station. The size of hexagons is determined by the spacing between sampling stations in the eye and the visual angle subtended by the image from the animal's viewpoint. For each sampling station, toBeeView calculates the weighted averages of the RGB values from the part of the image that the sampling station views. Finally, it colors the entire hexagon with the averaged RGB values calculated for its sampling station.

The amount of information available at the retina depends mainly on the density of photoreceptors and the optical quality of the eye. In the simplest configuration, toBeeView uses two parameters to characterize density and one to characterize optical quality. The angles ϕ_V_ and ϕ_H_, referred in what follows as vertical and horizontal interommatidial angles, are the angles separating consecutive sampling stations in the vertical and horizontal directions, respectively. They will be the angles between consecutive ommatidia in compound eyes and the typical angular distances between consecutive photoreceptors with the same spectral sensitivity in camera eyes. The optical quality of the eye is captured in the acceptance angle, ρ, which determines the area of the visual image projected onto each sampling station. Users must supply these parameters, as well as the vertical angle subtended by the image—all angles in degrees.

**Figure 1:**
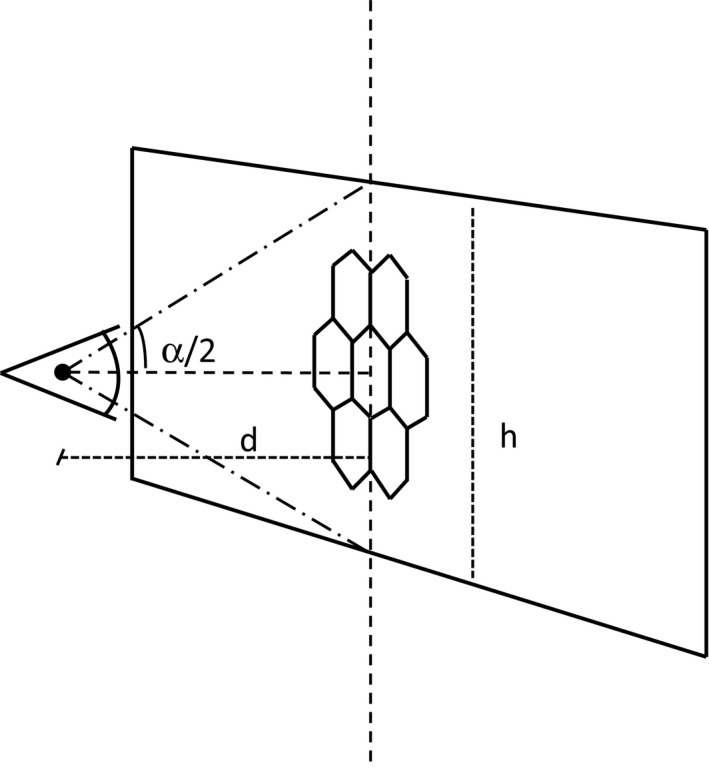
Relationship between the angle subtended by the visual scene, α, the photograph height, *h*, and the viewing distance, *d*

Let α be the vertical angle subtended by the visual scene at the insect's position. If the image has *h* rows of pixels, for all the calculations that follow we can assume that the insect is watching the photograph (not the scene, which need not lie on a single plane) from a distance *d*. This distance *d*, in pixel units, is (Fig. 1)

(1) $$d=\frac{h}{2\text{tg}(\alpha /2)}.$$

In terms of this distance, the vertical spacing between hexagons is 2*d*tg(ϕ_V_/2) and the horizontal spacing is 2·*d*·tg(ϕ_H_/2) (Spaethe & Chittka, 2003).

In the final image, each hexagon represents a sampling station in the animal eye and is assigned a unique homogeneous color. The hexagon color is calculated as a weighted average over all pixels on the image. Each hexagon is identified by a number *n* (*n* = 1, 2… number of hexagons). For the nth hexagon, the final color *C*_*n*_ is given by (Spaethe & Chittka, 2003)

(2) $$C_{n}=\frac{\sum \{\chi_{i}\text{exp}\left[-2.77{\left(\alpha_{i}/\rho \right)}^{2}\right]\}}{\sum \{\text{exp}\left[-2.77{\left(\alpha_{i}/\rho \right)}^{2}\right]\}},$$

where *C*_*n*_ is the color assigned to the nth hexagon, χ_*i*_ is the color of the pixel *i* and α_*i*_ is the angle between the segments joining the nodal point of the eye with the center of the hexagon *n* and the segment joining the nodal point of the eye with pixel *i*. The scanning mode assumes that the segment from the center of the hexagon to the eye is normal to the plane of the image. Thus, the output of toBeeView does not correspond to what a static viewer would see. Rather, it represents the information that the viewer could acquire scanning the image at a constant distance.

The RGB components are managed separately: toBeeView uses equation (2) three times for each hexagon, to calculate R_*n*_, G_*n*,_ and B_*n*_.

**Figure 2:**
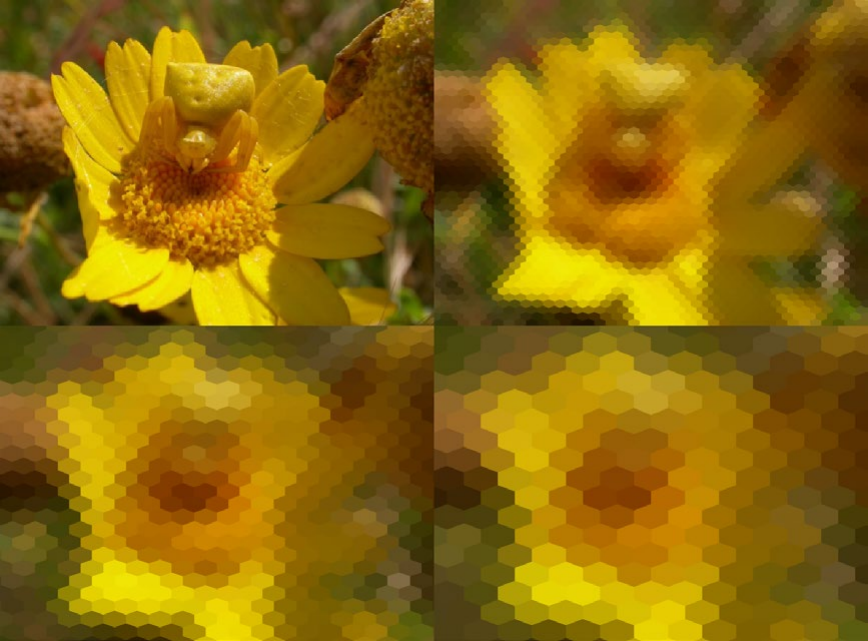
Female crab spider, *Thomisus onustus* (top left: photograph Eva de Mas) and projection on a honeybee retina at 5 cm (top right), 10 cm (bottom left), and 15 cm (bottom right). Scanning mode, basic chromatic configuration. Vertical and horizontal interommatidial angles are 0.9° and 1.6°; acceptance angle 2.6°

As an example, Fig. 2 shows a female crab spider *Thomisus onustus* Walckenaer (top left) and the amount of detail available to a honeybee *A. mellifera* L seeing the spider at a distance of 5 cm (top right), 10 cm (bottom left), or 15 cm (bottom right).

## 4 Color Treatment

When a single color photograph is analyzed as explained above, we ignore any mismatch between the sensitivity of the RGB channels and the photoreceptor types of our model animal. Typically, these mismatches are important (Stevens, Párraga, Cuthill, Partridge, & Troscianko, 2007)—particularly if we work with UV‐sensitive species. Furthermore, spatial acuity can differ between photoreceptor types (Giurfa, Vorobyev, Kevan, & Menzel, 1996).

**Figure 3:**
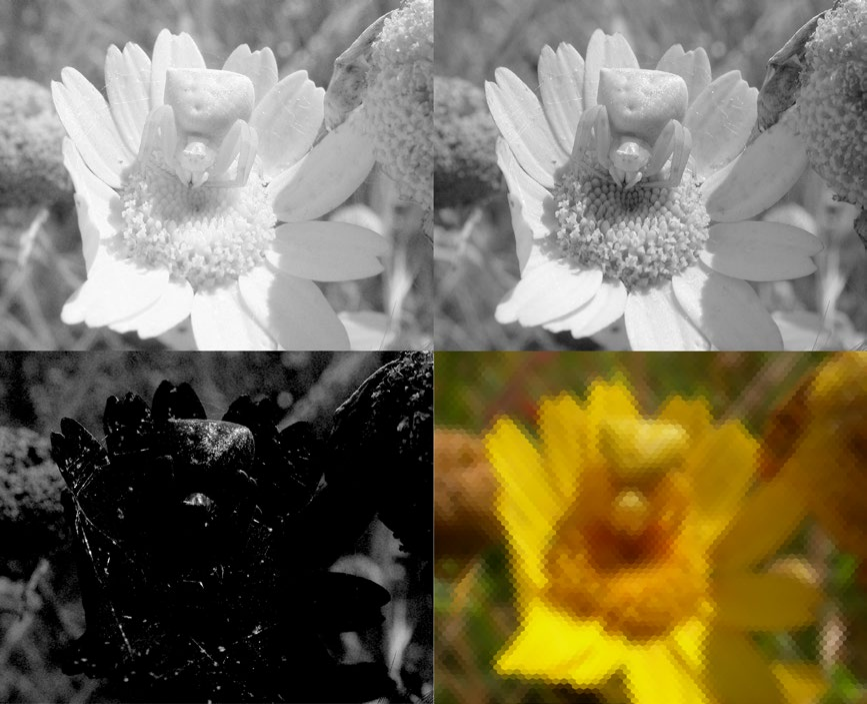
Female crab spider, *Thomisus onustus* of Fig. 2. R (top left), G (top right), and B (bottom left) channels of the original image, and toBeeView reconstruction of the image (bottom right). Scanning mode. Vertical subtended angle is 60°, corresponding to a distance of 3.5 cm from the spider. Vertical and horizontal interommatidial angles are 0.9° and 1.6°; acceptance angle 2.6°

To handle the first problem, we used the same approach as Vorobyev et al. (1997). Users can input a series of black and white photographs of the visual scene, each of them taken with a different filter, and specify a weighting matrix **W**, where *w*_*fc*_ is the weight of photograph *f* on color channel *c*. For each photograph *f* (*f* = 1, 2… up to the number of input photographs—with a maximum of nine), and hexagon *n*, toBeeView first computes *C*_*nf*_ using equation (2). (Note that, because the input files are in gray scale, the result is independent of whether the calculations are based on the R, G, or B channels.) toBeeView then combines these values to produce the color of the hexagon in the output image using matrix **W**. For instance, the value of the R channel in hexagon *n* would be

(3) $$R_{n}=\sum w_{fR}C_{nf},$$

where the sum is taken over all the input files, *f*. The choice of weight parameters will depend on the study species and the filters used (Chiao et al., 2009; Vorobyev et al., 1997). For illustration purposes, Fig. 3 shows the results of decomposing an image into its three R, G, B channels and reconstructing them with toBeeView using weights *w*_*fc*_ = δ_*fc*_ (equal to one for the file *f* corresponding to channel *c*, and to zero otherwise). Of course, in this case, we obtain the same results if we use the original photograph directly—the purpose of the figure is simply to illustrate the process. Examples of how insect chromatic perception differs from ours can be found elsewhere (Chiao et al., 2009; Vorobyev et al., 1997; Williams & Dyer, 2007).

**Figure 4:**
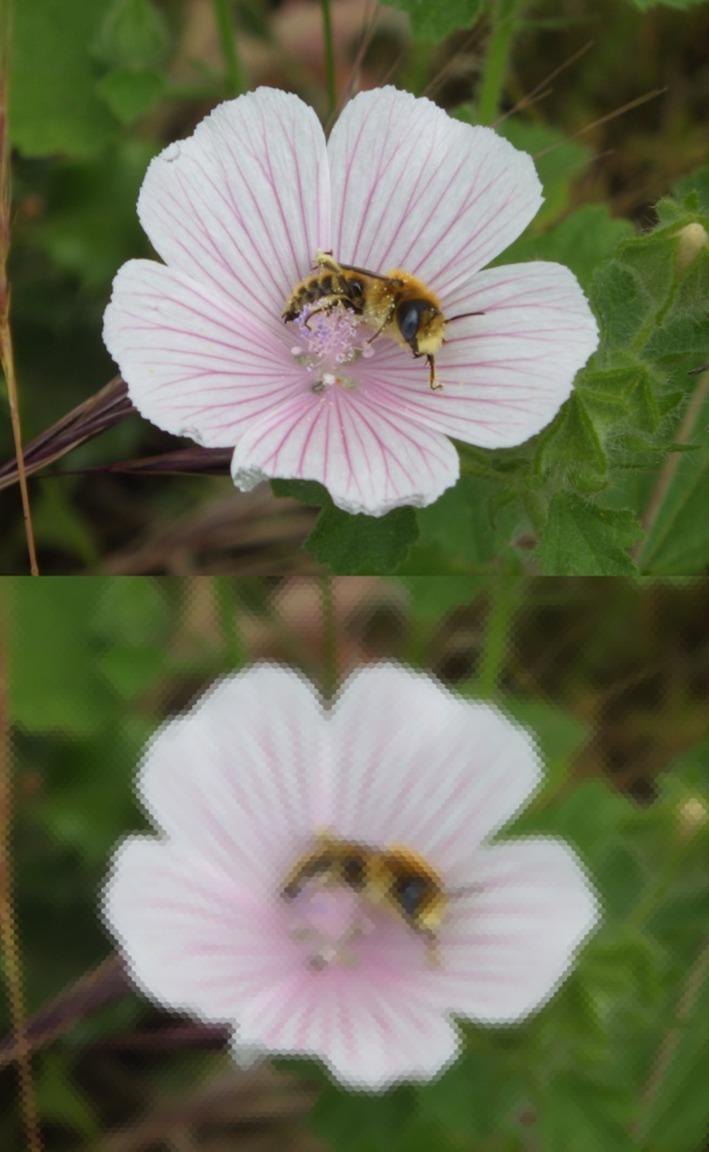
Pollen‐collecting bee (top) and reconstructed projection on the retina of an animal viewing the scene at a distance where the vertical subtended angle is 60° (bottom). Scanning mode, basic chromatic configuration. Vertical and horizontal interommatidial angles are 0.6° and 1.0° for the R channel and 0.9° and 1.6° for the G and B channels; acceptance angles are 1.3° for the R and 2.6 for the G and B channels

The second chromatic extension addresses the fact that different color channels may have different spatial resolution. Bees, for instance, detect objects offering green contrast at smaller subtended angles than objects with chromatic but no green contrast (e.g., Giurfa et al., 1996). The extent to which differences in photoreceptor densities and postretinal processing contribute to this result has not been worked out, but for many species, one photoreceptor type is more frequent than the others in the retina, and it is in principle possible that each color channel operates with different spatial parameters. Users can explore this possibility choosing different values for the interommatidial and acceptance angles of each chromatic channel. Figure 4 shows an example in which the spatial resolution of the R channel is greater than the resolution of the G and B channels. We suggest, however, that this exploratory tool is used with care.

## 5 Fixed‐viewpoint Mode

**Figure 5:**
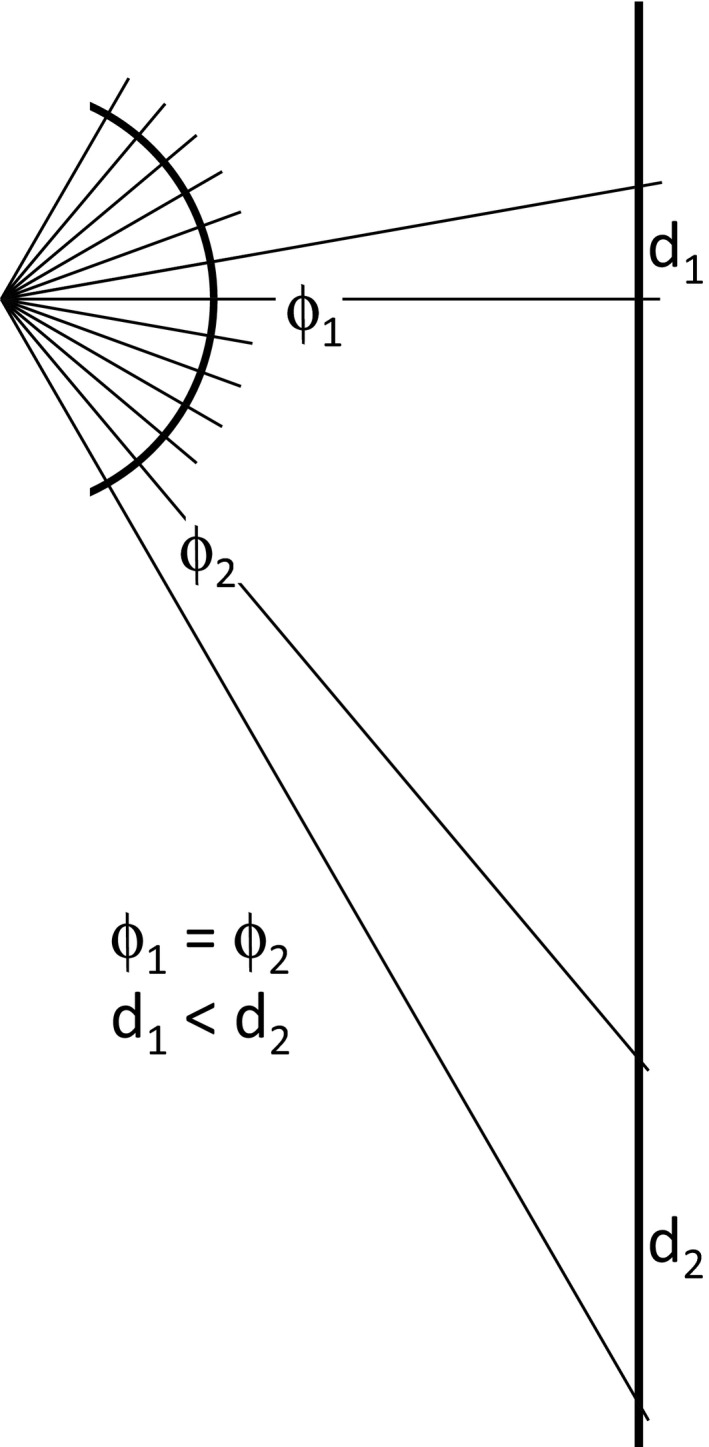
Distortion of the distance elements when a sphere is projected onto a plane

toBeeView superimposes a hexagonal grid on the image. In the examples so far, all the hexagons have the same size and shape. This is the same approach that has been used before (Giurfa et al., 1996; Spaethe & Chittka, 2003; Vorobyev et al., 1997), but it implies that the eye looks perpendicularly to the center of each hexagon—that is, that the viewer is scanning the image, making lateral movements to inspect it. It is easy to see why. If the scene is viewed by a stationary eye, the projection of the eye facets on the image is not a regular grid. The hexagonal eye facets tile a quasi‐spherical surface. Suppose that there is no regionalization, that the eye is composed of identical ommatidia and the angle between the axes of contiguous ommatidia is constant. Project the center of the facets on a plane (the plane of the image): The distance between consecutive centers is no longer constant (Fig. 5).

Users can choose to process the image as if the viewer were scanning it, flying at a constant distance of a plane on which the image is projected and staring perpendicularly to that plane, or looking at it from a fixed point. We now explain how the calculations are carried out when we assume that the viewer is looking from a fixed point.

When we look at the eye, we can select one “central ommatidium”: the one looking straight into the visual scene represented in the photograph. This ommatidum will be surrounded by a ring of six ommatidia, itself surrounded by a ring of 12 ommatidia, and so on. In general, the *n*‐th ring as we move outwards from the central ommatidium has 6·*n* ommatidia. The centers of these 6·*n* ommatidia, when projected onto the plane of the image, will lie on an ellipse with main axes on the vertical and horizontal directions of the plane. The center of the ellipse corresponds to the projection of the central ommatidium. In calculations, toBeeView assumes that projections of the hexagon centers are distributed regularly along the ellipse: To move from one projected center to the next along the ellipse, we must move a constant angle, 360/(6 *n*). The horizontal and vertical radii of the ellipse are calculated as follows. If the spacing of ommatidia in the eye is regular, then the radii *r*_H_ and *r*_V_ are easily calculated from the horizontal and vertical interommatidial angles:

(4) $$r_{H}=d\text{tg}\left(n\phi_{H}\right),$$

(5) $$r_{V}=d\text{tg}\left(n\phi_{V}\right),$$

where *d* is given by equation (1). In general, however, ommatidia are not evenly distributed. Interommatidial angles can change with eccentricity (angular distance from the central ommatidium). Users can therefore specify a quadratic relationship between interommatidial angle and eccentricity. The angle between rings m and *m* + 1 is

(6) $$\phi_{Xm}=\phi_{X0}+m\phi_{X1}+m^{2}\phi_{X2},$$

where X stands for H or V. Users must also input minimum and maximum values of ϕ_X*m*_. When interommatidial angles are not constant, *nf*ϕ_X_ in equations (4) and (5) must be replaced with ∑ϕ_X*m*_, where the sum goes from 0 to *n* − 1. Acceptance angles are likewise determined by a set of three user‐determined coefficients.

**Figure 6:**
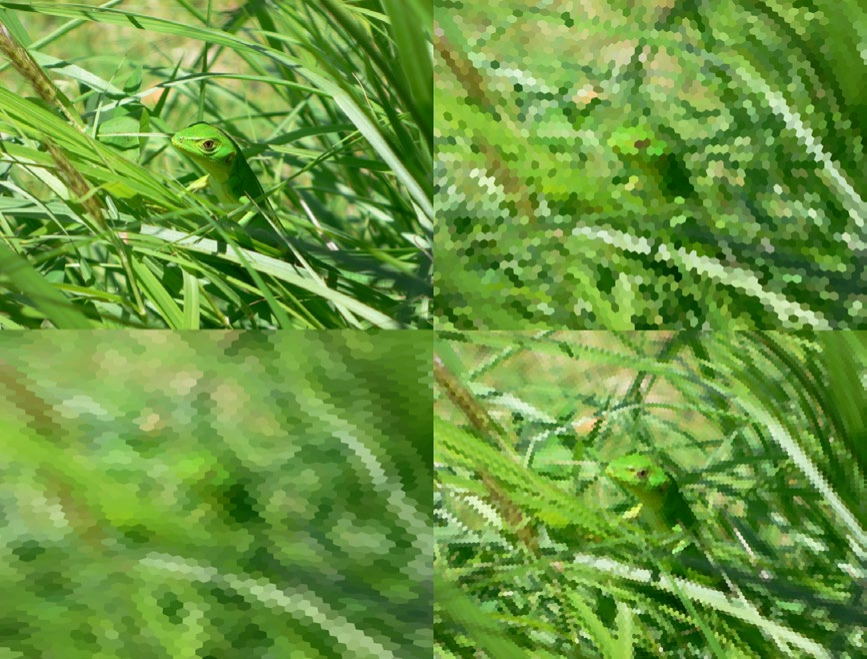
Lizard (top left) as viewed by a bee at 20 cm (bottom left) and a dragonfly at 40 cm (top right) or 20 cm (bottom right). Dragonflies have probably the best insect eyes, with spatial resolution close to 0.25° (Land, 1997). Human colors. Subtended vertical angles are 41.11° at 20 cm and 21.24° at 40 cm. For the bee view, vertical and horizontal interommatidial angles are 0.9° and 1.6°; acceptance angle 2.6°. For the dragonfly views, all angles are 0.25°

Once we have defined the projection of the ommatidia axes on the plane of the image, it is easy to determine the sides of the corresponding hexagons. The side separating a hexagon from one of its nearest neighbors is simply the perpendicular bisector of the segment joining their centers. The color of each hexagon is calculated as in equation (2), except that for the calculations of α_*i*_ we now assume that the eye is kept at a fixed position. Figure 6 shows a lizard as it could be seen by a stationary dragonfly or bee. The radial distortion introduced by the spherical shape of the eye is readily apparent in the figure, particularly at close distances with larger subtended angles and therefore more peripheral ommatidia involved.

## 6 Usage

toBeeView is a Win32 command line software with a single parameter: The name of a text file where all the relevant parameters are stored. As a result, toBeeView can be used in batch mode to process several images, or the same image with different eye geometries or scene distances. Because of the different options, however, it is easy to misformat the file. For this reason, although we specify in the Appendix how the file must be formatted, we have developed an interface that prompts users to input the different parameters, stores them with the proper format in a configuration file, and calls toBeeView. The two executable files (interface and program) must be in the same folder, and double‐clicking the interface application launches the process. The interface saves the configuration file in the same folder selected for the output image and with the same name as the output image (with “.txt“ extension). This way, users can easily check which parameters were used for each output image.

Valid input files are RGB images in BMP, JPG, TIFF, or PNG format. toBeeView does not accept images with indexed color schemes or alpha channel formats. Output will be in the same format as the input image.

toBeeView is distributed under GPL3 license. C# source files and a Win32 executable version can be found at https://github.com/EEZA-CSIC/compound-eye-simulator. The executable files can also be downloaded from http://www.eeza.csic.es/Pollination_ecology/

## 7 Modulation Transfer Function

To validate toBeeView, we can compare the visual acuity it predicts for a species with the one measured. Visual acuity can be explored with learning experiments. In a typical design, subjects are presented with two gratings with different orientation and obtain a reward when they approach the target orientation (for honeybees, see e.g., Srinivasan & Lehrer, 1988; Horridge, 2003). By presenting gratings with different spatial frequencies, and plotting the proportion of correct responses versus spatial frequency, we can see how response accuracy decreases as spatial frequency increases—the response versus spatial frequency function. For honeybees, the proportion of correct responses drops to 0.65 when the spatial frequency reaches approximately 0.25 cycles per degree (Horridge, 2003; Srinivasan & Lehrer, 1988).

The response versus spatial frequency function is tightly linked to the modulation transfer function of the eye, which we can study with toBeeView using as input an image of black and white stripes and changing the parameter values (subtended visual angle) to simulate changes in the spatial frequency of the pattern. We then select a band in the output image with a width of two periods (two stripes above and two below the central line), determine the maximum *I*_max_ and minimum *I*_min_ intensities within this band, and compute modulation as (Williams & Dyer, 2007)

(7) $$\text{Modulation}=\frac{I_{\max}-I_{\min}}{I_{\max}+I_{\min}}.$$

**Figure 7:**
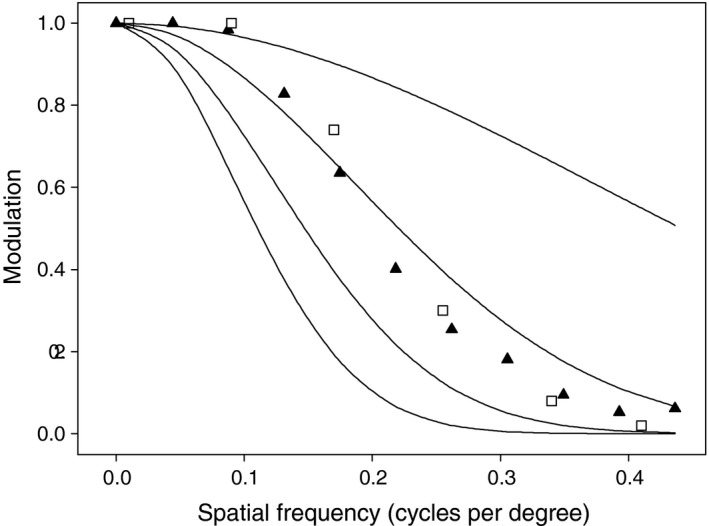
Simulated visual acuity in the honeybee. Modulation transfer function for the honeybee eyes as predicted by toBeeView (black triangles), and Gaussian modulation transfer function (solid lines; from bottom left to top right, ρ = 4, 3, 2, and 1). Empty squares represent the adjusted proportion of correct choices by honeybees discriminating between horizontal and vertical gratings (Srinivasan & Lehrer, 1988). To facilitate comparison, rather than plotting the proportion of correct choices (ranging from 0.5 under random choice to 1 with perfect discrimination), we plot 2·(proportion correct choices − 0.5), which ranges from 0 to 1 (data extracted from figure 3 of Srinivasan & Lehrer, 1988)

Figure 7 compares the modulation transfer function for the honeybee (ϕ_H_ =  1.6°,· ϕ_V_ = 0.9°, ρ = 2.6°; Laughlin & Horridge, 1971; Seidl, 1980) as predicted by toBeeView with the Gaussian modulation transfer function, derived from the formula

(8) $$\text{Modulation}=\text{exp}(-3.56{\left(\nu \cdot \rho \right)}^{2}),$$

where ν is the spatial frequency in cycles per degree (Srinivasan & Lehrer, 1988). We also plot the proportion of correct responses reported by Srinivasan and Lehrer (1988), which match very closely toBeeView's predictions.

## 8 Discussion

The output of toBeeView does not pretend to represent how nonhuman animals perceive visual scenes, but rather to estimate the amount of information that different visual systems may possess at the receptor stage when viewing a scene from a certain distance. In particular, there is no reason to assume that insects perceive the world as a mosaic of hexagonal tiles. Our retina, too, contains a finite set of receptors, but we do not perceive the visual field of each receptor as a spot. Information processing by the brain leads to a smooth, continuous perceived image (Dyer & Williams, 2005).

In a compound eye, each ommatidium can be approximated by a cone with hexagonal cross section. If we extend its walls forward, they will eventually intercept the visual scene represented on the input image, delimiting a hexagonal grid on the image. Near the center of the image, the spacing of rows and columns of hexagons may be 2*d*tg(ϕ_V_) and 2*d*tg(ϕ_H_/2), respectively, but as we move toward the periphery, the hexagons will become distorted and enlarged (Fig. 5). To make things worse, compound eyes are not spherical and interommatidial angles are not constant. The radii of curvature in the horizontal and vertical planes differ, and they change with eccentricity—there is often a relatively flat “acute zone” in the frontal area, and curvature increases in the periphery of the visual field (Collett & Land, 1975; Horridge, 1978). As a result, the ommatidia facets project on the image an extremely complex and highly species‐specific hexagonal grid.

Bees, however, use the frontal region of the eye for visual discrimination tasks (Srinivasan & Lehrer, 1988) and perform side‐to‐side scans over flowers when searching for ambushing predators (Gonzálvez & Rodríguez‐Gironés, 2013; Ings, Wang, & Chittka, 2012). For most applications, therefore, it may be most appropriate to use toBeeView in scanning mode, as in this mode, the output of the program provides the information that an insect would have after scanning the image with side‐to‐side scans. Users can, however, choose the fixed‐viewpoint mode and include some degree of heterogeneity in ommatidia size by specifying a quadratic relationship between interommatidial angle and distance from the center. More complex geometries cannot be implemented in toBeeView, but we doubt whether this is a problem: The periphery of the eye is mainly devoted to tasks such as motion detection or polarization measurement (Cronin, Johnsen, Marshall, & Warrant, 2014).

Unlike compound eyes, many camera eyes do not have their receptors regularly spaced. The hexagonal arrangement of toBeeView does nevertheless constitute a useful approximation to real cone arrangements in vertebrate eyes (Kram, Mantey, & Corbo, 2010) and maximizes visual acuity for a given photoreceptor density (French, Snyder, & Stavenga, 1977; Manning & Brainard, 2009). As for compound eyes, the visual acuity of camera eyes changes throughout the retina. Most camera eyes have a specialized region (called fovea in vertebrates) with maximum acuity (Cronin et al., 2014). In our opinion, toBeeView should be used to study the image projected on the fovea, using the scanning mode except when the angle subtended by the image is small enough that the entire image projects on the fovea, in which case the fixed‐viewpoint mode can be used.

When selecting values for the “interommatidial” angles, users should remember that these parameters refer to the angles separating photoreceptors with the same spectral characteristic. These will typically be larger than the inter‐receptor angles reported in studies of the visual system and can differ between receptor channels—for instance, in insects long‐wavelength photoreceptors are more abundant than short‐ and medium‐wavelength receptors (Arikawa, 2003; Hardie, 1986; Schwind, Schlecht, & Langer, 1984; Wakakuwa et al., 2005). toBeeView allows users to explore the consequences of this variability using different interommatidial angles for the R, G, and B channels.

The ability of toBeeView to capture the spatial resolution of insect eyes is validated by the tight fit between its predicted modulation transfer function and honeybee's choices in a discrimination test (Fig. 7). Its accuracy to represent perceived colors is limited by user input. In its simplest chromatic configuration, toBeeView follows the same RGB system of the original input image. Users requiring more complex chromatic treatment must first take a series of gray scale pictures, with different filters, and determine the weighting matrix **W** that best suits their set of filters and study system. This exercise can be performed using standard band‐pass filters (Vorobyev et al., 1997) or with custom‐made filters that mimic the sensitivity profile of the photoreceptors (Chiao et al., 2009). While the methodology we use is known to be reliable (Chiao et al., 2009; Vorobyev et al., 1997), the soundness of the process depends entirely on proper calibration of the equipment (Stevens et al., 2007).

To conclude, the output of toBeeView can be used to assess the amount of information present in the retina of an eye. This knowledge can be important to answer ecological and evolutionary questions. Thus, in Fig. 2, we can use toBeeView to estimate at what distance, a honeybee visiting flowers might be likely to detect the presence of the spider. More generally, we can use toBeeView to investigate which traits can be used as signals or can facilitate camouflage, taking into consideration the visual system of our study species.

## Funding Information

Spanish Ministerio de Ciencia e Innovación (Grant/Award Number: CGL2015‐71396‐P).

## Conflict of Interest

None declared.

## Data Accessibility

Source code and program can be downloaded from https://github.com/EEZA-CSIC/compound-eye-simulator.

## Appendix 1

### 1.1

The configuration file must contain the following information:

First line: number of input files to be processed, *n*

Lines 2 to *n* + 1: name of input files (full path) and weights associated with that file. Within lines, all parameters are separated by semicolons. Weights appear in the order R, G, B.

Next line: vertical angle subtended by the image at the viewer's eye.

Next line: viewpoint. Use 0 for scanning mode and 1 for static viewer.

Next line: number of grids. Use 0 for one grid, 1 for three grids (one per color channel).

Next three lines: interommatidial and acceptance angles (in degrees). One line for the horizontal interommatidial angles, one for the vertical, and one for the acceptance angles. For each line, type three values (corresponding to the S, M, and L wavelength channels) or if all the channels have the same resolution type a single value followed by two semicolons.

Next four lines: coefficients of the relationship between eccentricity and interommatidial angle. Linear coefficient for the horizontal angle, linear coefficient for the vertical angle, and quadratic coefficients. For each coefficient, three values (S, M, and L wavelength channels) or one value followed by two semicolons. If you have selected the scanning mode, each of these four lines must contain just two semicolons.

Next two lines: maximum and minimum values of the interommatidial angles (in degrees).

Last line: name of output file.
